# Sex Differences in Aggressive Traits and Superior Temporal Cortical Responses to Monetary Loss

**DOI:** 10.3390/bs16101778

**Published:** 2026-09-29

**Authors:** Guangfei Li, Yingjie Cao, Yongxiu Yang, Xinyu Zhang, Bao Li, Hao Sun, Suqin Huang, Mengdi Gao, Guangyu Bin, Chiang-Shan R. Li

**Affiliations:** 1 Department of Biomedical Engineering, College of Chemistry and Life Science, Beijing University of Technology, Beijing 100021, China; 2 Department of Psychiatry, School of Medicine, Yale University, New Haven, CT 06520, USA; 3 Department of Neuroscience, School of Medicine, Yale University, New Haven, CT 06520, USA; 4 Interdepartmental Neuroscience Program, School of Medicine, Yale University, New Haven, CT 06520, USA

**Keywords:** reward, punishment, aggression, sex differences, fMRI

## Abstract

*Background*: Individuals vary in aggression traits. Here, we investigated how individual aggression is associated with altered reward and punishment processing in the brain and the neural bases of sex differences. *Methods*: Aggression was assessed by the Achenbach Adult Self-Report. We curated the Human Connectome Project dataset and modeled the BOLD signals to identify regional responses to reward and punishment blocks in the gambling task (n = 981, 473 men) as well as responses to the identification of negative emotional face in a target-matching task (n = 885, 436 men). Whole-brain statistical significance was assessed at the voxel-level *p* < 0.001 (uncorrected) with cluster-level family-wise error (FWE) correction at *p* < 0.05. For regional responses identified in men or women alone, we conducted slope tests to examine the sex differences in their relationships with individual aggression. *Results*: Whole-brain regression showed higher activation of the left superior temporal gyrus (STG) during gambling loss in men. We extracted the left STG response to reward versus baseline (STG-win β) and to loss versus baseline (STG-loss β), where β is the GLM contrast estimate. Left STG-loss β was significantly correlated with aggression score in men but not in women, and a slope test confirmed the sex difference (*Z* = 3.54, *p* < 0.001). STG-loss β but not STG-win β was significantly correlated with aggression score in men, and a slope test confirmed the specificity of this valence-related correlation (*Z* = 3.66, *p* < 0.001). Further, the correlations of STG-loss β and of STG response to negative emotional faces versus shapes with aggression score differed in men (*Z* = 2.70, *p* = 0.007). *Conclusions*: STG response to loss represents a neural correlate of aggression in men but not in women. The STG-loss response did not reflect a broader relationship between aggression and negative emotion.

## 1. Introduction

### 1.1. Human Aggression

Aggression represents a mental state or behavior intended to cause harm in another (Liu, 2004). Studies of the evolutionary origin of human behavior link autonomic “fight or flight” responses to the outward expression of aggression (Archer, 2009). Human aggression can be reactive or proactive, with the latter associated with the evolution of groupishness and social norms (Sarkar & Wrangham, 2023). Although typically studied as psychopathology that conduces violence and criminal behavior, aggression represents a broader construct that includes physical, verbal, and other psychological means to cause harm. Aggression may emerge in childhood and adolescence and progress throughout adulthood. Children exposed to family violence exhibited aggressive behavior more frequently (Doroudchi et al., 2023). Aggression is associated with less empathy and higher callous-unemotional traits, suggesting dysfunctional emotional processing in adolescents (Ritchie et al., 2022). Other studies highlighted the roles of emotional lability in aggressive acts among people with high negative urgency (Dvorak et al., 2013). Many have also investigated the biological, including genetic, bases of aggression (Zhang-James & Faraone, 2016) and implicated the monoaminergic circuits (Ferrari et al., 2005) as well as gene–environment interaction (Cupaioli et al., 2021; Kolla & Bortolato, 2020) in the manifestation of aggression.

### 1.2. Reward/Punishment Processing and Aggression: Behavioral Evidence

Reward and punishment sensitivity are critical to individual differences in behavior (Jonker et al., 2022). Gray’s Reinforcement Sensitivity Theory conceives reward and punishment sensitivity integral components of our personality, with the behavioral approach system (BAS) and behavioral inhibition system (BIS) each involving conditioned responses to appetitive and aversive stimuli. The BAS drives behavior toward reward and the BIS suppresses actions associated with punishment (Balconi et al., 2009; Barrós-Loscertales et al., 2010; Megías-Robles et al., 2022). In their extremes, BAS and BIS reactivities may contribute to various forms of psychopathology, including substance use disorder (Che et al., 2020), attention deficit hyperactivity disorder (J. J. Li, 2018), depression (Kasch et al., 2002; Sun et al., 2020) and anxiety (Takahashi et al., 2021), and aggression (Bjørnebekk, 2007; Constantinou et al., 2011; Parker et al., 2022).

In college students, reactive, but not proactive, aggression was significantly associated with the BIS, whereas both forms of aggression were significantly associated with the BAS (Pederson et al., 2018). In another study, participants were subject to insults by another person and, following priming with a neutral or approach-motivating event, asked to make a hiring recommendation about this other person, as a way to evaluate aggression. The results showed that individuals with greater approach motivation, i.e., higher BAS traits, are more likely to show aggression in their decision (Harmon-Jones & Peterson, 2008). In another study that combined recording of skin conductance response (SCR) and a reward learning paradigm, children high in proactive aggression showed reduced SCR to cues predicting rewards (Gao et al., 2018). Thus, although the findings varied, the studies appeared to implicate both BAS, or reward sensitivity, and BIS, or punishment sensitivity in human aggression. On the other hand, extant studies employed largely questionnaire assessments and have not examined neural responses to reward or punishment in relation to individual aggression.

### 1.3. Imaging Studies of Aggression

A review of the neurobiology of human aggression highlighted changes in regional brain volumes, metabolism, and connectivity in a wide swath of brain regions, including the prefrontal cortex (PFC), insula, amygdala, basal ganglia and hippocampus (Cupaioli et al., 2021). The great majority of studies of aggression examined regional responses to unfair offers in social interaction and/or to retaliatory actions. For instance, adolescents with bipolar disorder showed higher activity of the left subgenual anterior cingulate cortex (ACC), right amygdala, left orbitofrontal cortex (OFC), and right thalamus during frustrative non-rewards—correct responses deemed “too slow” and disqualified for reward—and these regional activities were inversely correlated with aggression (Barzman et al., 2014). In college students undergoing imaging during a behavioral aggression task, provocation led to greater activity in the ventral striatum (VS), which predicted more vigorous retaliation in the task and participants’ history of real-world violence. Notably, the functional connectivity between the VS and lateral PFC was associated with lower retaliatory aggression, suggesting a regulatory role of the PFC (Chester & DeWall, 2016). Investigating whether aggressive behavior may be intrinsically rewarding, a more recent work engaged men in the video game Carmageddon during brain imaging and showed that non-violent and violent success elicited activation of the VS and dorsal striatum, respectively (Klasen et al., 2020). The studies together highlighted the neural processes of reactive aggression during social exchange, which often involves complex behavioral interaction. While studies have associated the BAS with higher activation in bilateral VS during monetary wins and of the right lateral OFC during losses (S. H. Kim et al., 2015), it remains less clear whether the neural processes of reward or punishment receipt may vary with individual aggression. Although most imaging studies have focused on reactive aggression during social exchange, a few studies have examined reward and punishment processing in relation to aggression. For example, boys with conduct problems showed altered neural responses during a gambling task (Schwenck et al., 2017), and appetitive motivation predicted neural responses to facial signals of aggression (Beaver et al., 2008).

### 1.4. Sex Differences in Aggression and Reward/Punishment Processing

Sex plays a critical role in aggressive behaviors (D. Kim et al., 2022; Lussier et al., 2012), which may be influenced by sex-specific neurosteroids (Carré et al., 2009; de Almeida et al., 2015; Geniole et al., 2020; Hilz & Lee, 2023; Yildirim & Derksen, 2012) and, as shown in young children, environmental factors (Baillargeon et al., 2007). Boys are overall more aggressive than girls, and boys and girls are each more physically and psychosocially aggressive (Björkqvist, 2018). The associations between negative emotions and aggression appeared stronger for males than females (Chen et al., 2012).

Imaging studies, including those described above, have not specifically examined sex differences in the neural processes of aggression, despite findings from animal studies (Z. Zhu et al., 2024). On the other hand, there is abundant evidence for sex differences in reward and punishment processing. For instance, monetary gain relative to loss induced stronger increases in VS dopamine synthesis in men, whereas the opposite was true in women, and these differences were associated with reward and punishment sensitivity in men and women, respectively (Hahn et al., 2021). One study combined electroencephalography and a guessing task and showed that boys were less likely to switch their response after punishment and had less feedback-related negativity (FRN) than girls (Ding et al., 2017). A study that included probabilistic punishment (foot shock) in a reward-seeking task reported a greater increase in response latency in females than in males, suggesting a more prominent influence by risk of punishment in reward seeking (Chowdhury et al., 2019). A few studies have begun to address sex differences in the neural processes of aggression. For instance, Herpertz et al. reported sex-specific brain mechanisms underlying reactive aggression in borderline personality disorder (Herpertz et al., 2017). In a modified Taylor Aggression Task, men showed higher left amygdala activation during provocation than women (Repple et al., 2018). Moreover, our previous work has shown sex differences in the neural correlates of anger traits and negative facial emotions (G. Li et al., 2020a) and in ventral striatal responses to monetary loss in relation to externalizing traits (G. Li et al., 2023b). Importantly, these sex differences in reward and punishment processing may overlap with the neural mechanisms of aggression. The ventral striatum and amygdala, which are central to reward and punishment processing, have also been implicated in reactive aggression in a sex-dependent manner (Herpertz et al., 2017; Repple et al., 2018). These overlaps suggest that sex differences in aggression may partly arise from sex differences in how the brain processes reward and punishment.

### 1.5. The Present Study

However, few studies have directly examined reward and punishment responses in relation to individual aggression within the same participants, and the role of sex in this relationship remains unclear. Here, we employed the Human Connectome Project (HCP) data collected of a guessing task in young adults and tested the hypothesis of a higher correlation between regional responses to reward and/or punishment and aggressive trait in men than in women.

## 2. Materials and Methods

### 2.1. Study Design and Setting

This study was a secondary, cross-sectional analysis of de-identified data from the Human Connectome Project (HCP) S1200 release. No new participants were recruited, and no new data were collected for the present study. The original HCP study was conducted in accordance with the Declaration of Helsinki and was approved by the Washington University Institutional Review Board (IRB #201204036). The present analyses used 3T MRI scans and behavioral task data collected in young adults.

### 2.2. Data Sources and Measurement

Data were obtained from the HCP S1200 release, which comprises 3T MRI scans and gambling task data from 1080 participants (G. Li et al., 2023a, 2023c, 2023d; Van Essen et al., 2012). Aggression was measured using the Achenbach Adult Self-Report (ASR) Syn-drome Scales (Achenbach, 2009). The aggressive behavior subscale comprises 15 items, each scored from 0 to 2, with higher scores indicating more aggression problems (Supplementary Methods). Imaging tasks were acquired as part of the HCP protocol. Participants underwent two runs of a gambling task (~3 min 12 s per run), each consisting of four blocks (two reward and two punishment conditions, with win/loss trials), interspersed with 15 s fixation periods (Barch et al., 2013). To assess generalization to motivational salience under negative emotional exposure, we additionally analyzed data from a face identification task (available for n = 885/981; 436 males). In this task, participants matched target images during blocks featuring negative emotional faces (angry, fearful) or ellipse shapes (G. Li et al., 2020a). Detailed imaging protocols and task procedures are provided in the Supplementary Methods (G. Li et al., 2021, 2023b).

### 2.3. Participants and Sampling

The HCP S1200 release is a community-based sample enriched for twins and siblings and is not a population-based probability sample. All participants were free of major neurodevelopmental, psychiatric, or neurological conditions. Following quality control, 99 of the 1080 participants were excluded because of excessive head motion (>2 mm translation or >2° rotation) or failed image registration. No additional exclusion criteria were applied. The final analyzable sample comprised 981 participants (473 males, mean age 27.9 ± 3.6 years; 508 females, mean age 29.6 ± 3.6 years). Given the significant age difference between sexes, age was treated as a covariate in all analyses. Demographic and aggression data are summarized in Table 1.

As this was a secondary analysis of an existing cohort, no a priori sample size calculation was performed. The analyzable sample size was determined by the HCP S1200 release and image quality control. Conventional sampling error was not formally quantified; however, family relatedness was accounted for in sensitivity analyses using generalized estimating equations (GEE).

### 2.4. Variables

The primary variable of interest was the continuous ASR aggression score. Imaging variables included regional BOLD responses to monetary win versus baseline (STG-win β), monetary loss versus baseline (STG-loss β), and negative emotional faces versus shapes (STG-NegEmo β). Covariates included age and sex. The key interaction term was sex × centered aggression score.

### 2.5. Bias

Several potential sources of bias were considered. Head motion and image registration failure were addressed through quality control. Selection bias may be present because the HCP sample is neurotypical and not clinically ascertained. Information bias may arise from self-reported aggression. Circular inference in the functionally defined left STG ROI was addressed using an independent anatomical ROI from the SPM Anatomy Toolbox. Non-independence due to family relatedness was addressed using GEE with family ID as the clustering variable. Finally, the gambling and face-processing tasks differ in stimulus type, cognitive demands, and outcome contingencies; therefore, comparisons between loss processing and negative emotion processing were interpreted cautiously.

### 2.6. Study Size

The final sample included 981 participants (473 men, 508 women). The face identification task was available for 885 participants (436 males). As noted above, the sample size was fixed by the HCP S1200 release and quality control procedures; no formal power calculation was performed for this secondary analysis.

### 2.7. Statistical Methods

Image preprocessing and analysis followed established methods (G. Li et al., 2020b). BOLD signal modeling identified regional responses to: (1) reward/punishment blocks versus baseline in the gambling task, and (2) negative emotional faces versus shapes in the matching task (Supplementary Methods). Statistical significance was assessed at voxel-level *p* < 0.001 (uncorrected) with cluster-level FWE correction (*p* < 0.05) using SPM’s Gaussian random field theory implementation. The subsequent ROI correlation, slope tests, valence comparison (loss vs. win), and negative emotion comparison were descriptive/illustrative and involved a small number of pre-specified tests; we therefore report exact *p*-values, effect sizes, and 95% CIs without additional correction.

Whole-brain regression analyses examined aggressive behavior associations in the full cohort (covariates: age, sex) and sex-specific subgroups (covariate: age). Regional activity was extracted using MarsBar (http://marsbar.sourceforge.net/, accessed on 9 October 2019) for individual-level analysis: the activity (β contrasts averaged across voxels) of win vs. baseline (STG-win β) and loss vs. baseline (STG-loss β). β refers to the general linear model parameter estimate for a given contrast (e.g., loss vs. baseline). More positive values indicate relatively greater regional BOLD response to the condition of interest relative to the reference condition; negative values indicate relatively lower response.

To formally test sex differences, we conducted a hierarchical regression with STG-loss β as the dependent variable. Age, sex, and centered aggression score were entered in Step 1; the sex × centered aggression interaction was entered in Step 2. Simple slopes were then examined to confirm sex difference and valence specificity. Further, because negative emotion is also implicated in aggression, we also computed the STG β estimates of “face—shape” blocks (STG-NegEmo β) in the face identification task.

To address potential circularity in the functionally defined left STG ROI, we performed an independent anatomical validation using the SPM Anatomy Toolbox. The left STG ROI was defined by the probabilistic cytoarchitectonic map Anatomy3_99_1Auditory_Te11 (left An99), and the right STG ROI was defined by Anatomy3_100_rAuditory_Te11 (right An100). For each participant, mean β estimates for loss vs. baseline were extracted from these anatomical ROIs using MarsBar. A generalized estimating equation (GEE) model with family ID as the clustering variable was used as a sensitivity analysis to account for non-independence among related participants.

## 3. Results

### 3.1. Clinical and Gambling Task Performance Measures

Aggression score showed significant sex difference, with men showing higher scores than women (Table 1).

As previously reported (G. Li et al., 2023b), punishment blocks elicited shorter RTs than reward blocks, and men showed shorter RTs than women. The statistics of ANCOVA are shown in Supplementary Table S1. RT measures (reward, punishment, post-loss, post-win, and difference scores) were not correlated with aggression score in the full sample, men, or women (all *p*’s > 0.104; see Supplementary Results). Thus, no performance metrics of the gambling task could serve as a marker of aggression.

### 3.2. Regional Activations to Win and Loss in Correlation with Aggression Score

Whole-brain analyses revealed no significant associations between reward-related activation (reward—baseline) and aggression scores at the specified threshold (voxel *p* < 0.001 uncorrected, cluster *p* < 0.05 FWE-corrected), either in the full cohort or in sex-specific subgroups.

**Figure 1:**
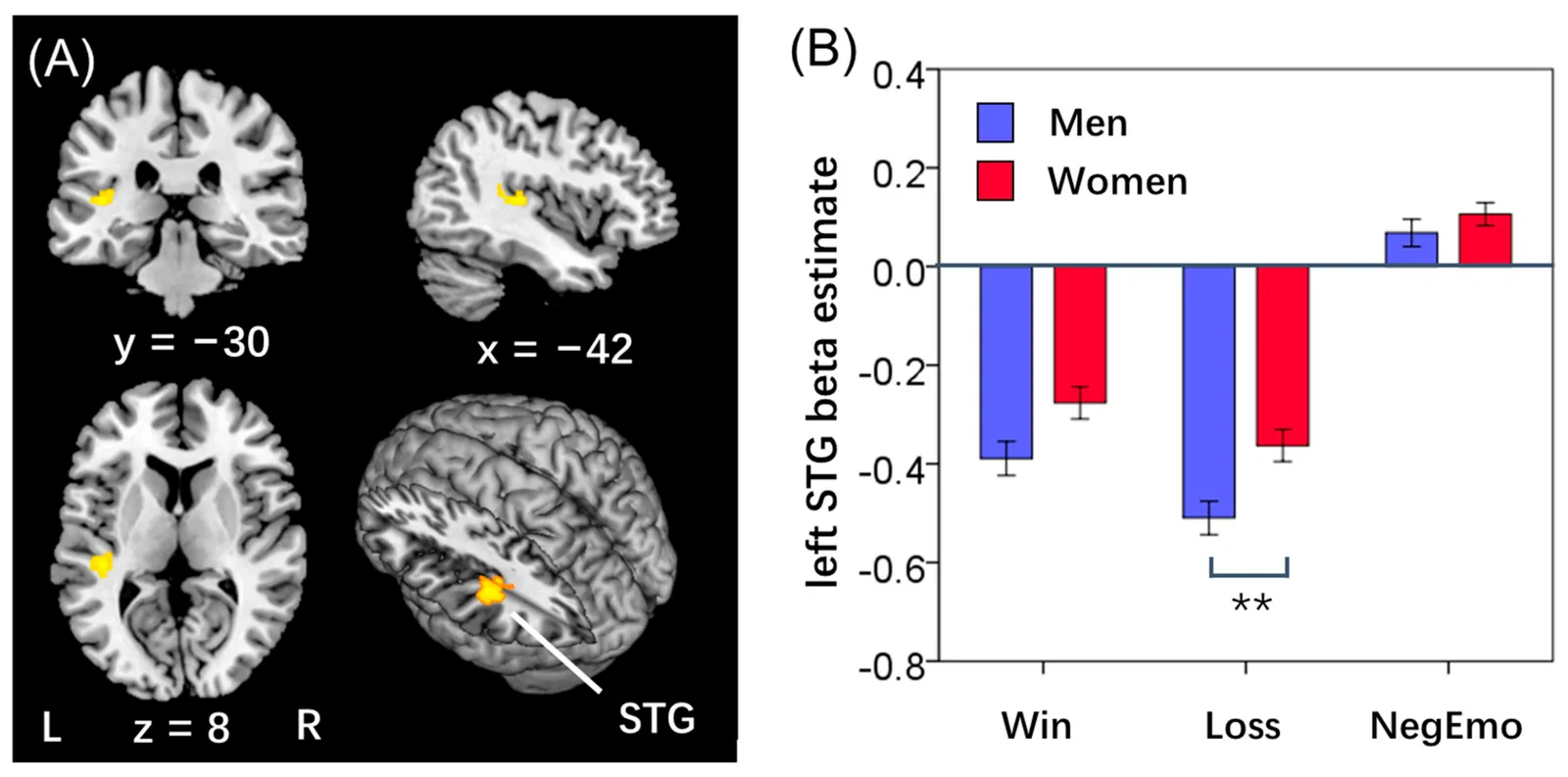
(**A**) STG mask. (**B**) STG response (β) to reward (STG-win), punishment (STG-loss) and negative emotion (STG-NegEmo) in men and women. ** *p* < 0.01.

For the contrast “punishment—baseline”, whole-brain linear regression against aggression score for the entire sample or for women alone with the same covariates revealed no clusters at the same threshold. In men alone, a cluster in the left STG (x, y, z = −42, −30, 8; *T* = 4.55, 864 mm^3^) showed a positive correlation with the aggression score (Figure 1A).

Our previous study of a slightly different sample of the HCP reported the results of a one-sample *t* test of “reward—baseline” and “punishment—baseline,” with the left STG showing significantly lower response (“deactivation”) to both reward and punishment relative to baseline (G. Li et al., 2020b). Here, we computed the β estimates (STG-win and STG-loss β’s) for all subjects to visualize these findings. Further, to examine sex differences, we performed a valence × sex repeated measures ANCOVA, and the results showed no significant valence (*F* = 0.59, *p* = 0.444) or sex (*F* = 2.41, *p* = 0.121) main effect, nor valence × sex interaction effect (*F* = 0.74, *p* = 0.389). In post hoc comparisons, STG-win β did not show significant sex difference (*t* = −1.81, *p* = 0.070), but STG-loss β was significantly lower in men than in women (−0.49 ± 0.73 vs. −0.36 ± 0.68; *t* = −2.77, *p* = 0.006, Figure 1B).

To examine whether the loss-related response of the STG may reflect negative emotion processing, we also computed the β estimates of “face—shape” of the STG (STG-NegEmo β’s) for individual subjects. Men (0.07 ± 0.58) and women (0.11 ± 0.50) did not differ significantly in STG-NegEmo β (*t* = −1.05, *p* = 0.293; Figure 1B).

### 3.3. STG Responses to Win/Loss: The Influences of Aggressive Trait in Men

**Figure 2:**
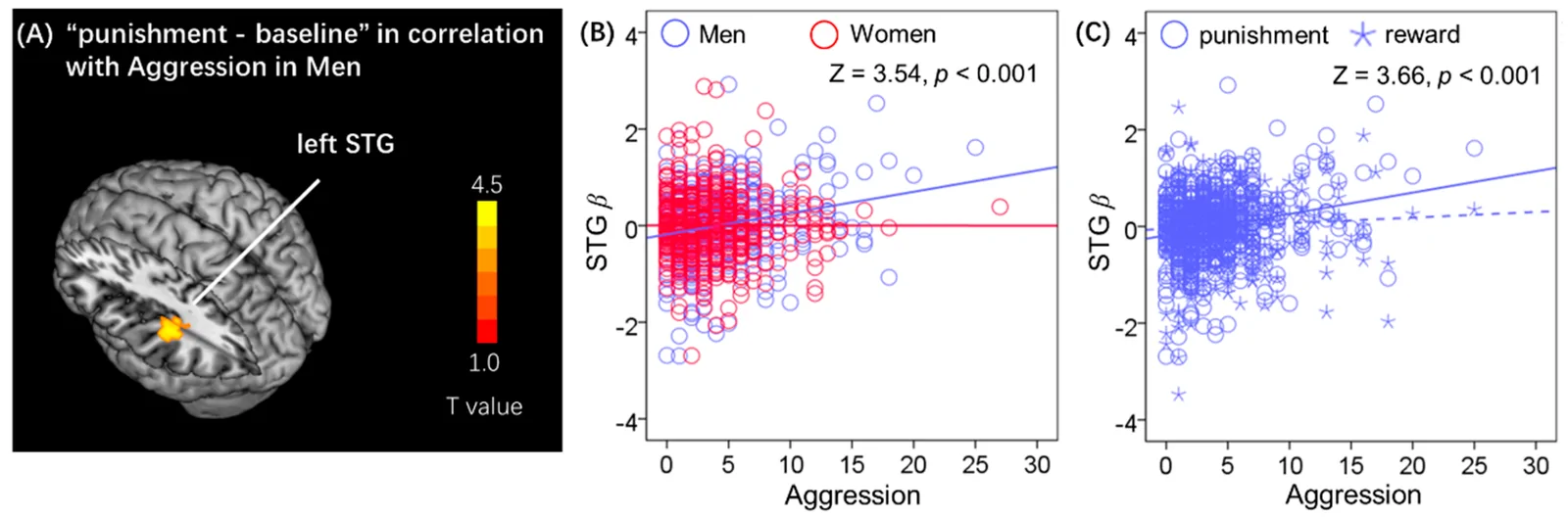
(**A**) In men only, left STG response to punishment showed positive correlation with aggression score. (**B**) Scatter plot of the β estimates of left STG (STG β) vs. aggression score in men (solid blue line) and in women (solid red line); slope test confirmed the sex difference. (**C**) Scatter plots of STG response to punishment (STG-loss β) and to reward (STG-win β) vs. aggression score in men; the solid and dashed lines represent linear regression fits for punishment and reward, respectively slope test indicated that this aggression-related STG response was relatively specific to monetary loss. Note that the residuals are plotted here with age accounted for.

The β estimate of the left STG (STG β, cluster shown in Figure 2A) was significantly correlated with aggression score (*r* = 0.22, CI [0.13, 0.30], *p* < 0.001; Figure 2B) in men, as expected, but not in women (*r* = 0.00, CI [−0.09, 0.09], *p* = 0.998). We confirmed the sex difference with a slope test (*Z* = 3.54, CI [0.10, 0.35], *p* < 0.001). These follow-up ROI analyses are descriptive rather than confirmatory, because the functional left STG ROI was defined from the aggression-related whole-brain regression in men. To mitigate circular inference, we validated the key association using an independent anatomical left STG ROI.

In a unified interaction model, the sex × aggression interaction on STG-loss β was significant, ΔR^2^ = 0.012, F = 11.80, *p* = 0.001; B = 0.044, SE = 0.013, t = 3.44, *p* = 0.001, CI [0.019, 0.069]. Simple slopes showed a significant positive association in men (B = 0.044, *p* < 0.001) but not in women (B = 0.000, *p* = 0.972). These results confirm that the association between aggression and STG-loss β differs by sex.

Further, for men alone, slope test was used to examine the difference between the correlation for each of STG-win β and of STG-loss β vs. aggression score; the result showed significant difference in men (*Z* = 3.66, *p* < 0.001, Figure 2C). This pattern suggests a relative specificity to monetary loss rather than reward in men. Because both contrasts were derived from the same gambling task, this should be interpreted as relative rather than absolute specificity.

To assess whether the main finding depended on the functionally defined ROI, we repeated the key analyses using independent anatomical left and right STG ROIs from the SPM Anatomy Toolbox. In men, left An99 STG-loss β was positively correlated with aggression score (*r* = 0.17, *p* < 0.001, Supplementary Figure S1). The association was not significant in women (*r* = 0.00, *p* = 0.997). We confirmed the sex difference with a slope test (*Z* = 2.73, *p* = 0.006). These results indicate that the association between aggression and STG-loss response is not dependent on the functionally defined ROI and is unlikely to be driven by circular inference.

To account for the non-independence of related participants (siblings and twins) in the HCP dataset, we re-ran the key interaction analysis using generalized GEE with family ID as the subject variable and an independent working correlation matrix. Consistent with the hierarchical regression results, the sex × aggression interaction on STG-loss β remained significant (B = 0.044, SE = 0.013, CI [0.018, 0.069], Wald χ^2^ = 11.11, *p* = 0.001). Simple slopes indicated a significant association in men (B = 0.044, *p* = 0.001) but not in women (B = 0.000, *p* = 0.968). These results confirm that the sex difference is robust to family relatedness.

### 3.4. Loss Processing vs. Negative Emotion Exposure

To assess whether STG responses to loss reflect generalized negative valence processing, we analyzed the HCP face identification task data. For each participant, we extracted β estimates for negative emotional faces versus shapes (STG-NegEmo β), representing neural responses to negative affect. These values were then regressed against aggression scores in the full cohort and sex-specific subgroups, mirroring our loss-response analyses.

**Figure 3:**
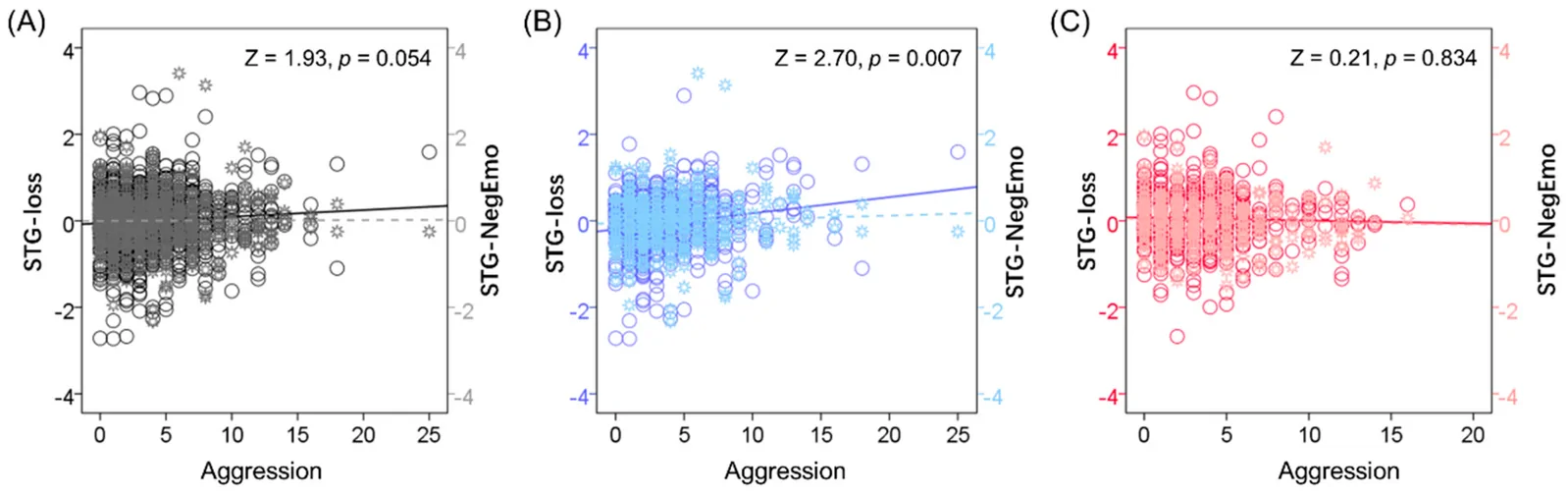
Linear regression of STG response to punishment (STG-loss β, circles, dark color and solid line) and to negative emotional face identification (STG-NegEmo β, asterisks, light color and dashed line) vs. aggression score: in (**A**) all (black), (**B**) men (blue), and (**C**) women (red). *Z* and *p*-values indicate regression slope differences (age-adjusted residuals shown).

STG-NegEmo β was not correlated with aggression score in the full cohort or sex-specific subgroups (all *p*’s > 0.294). The correlation of STG-loss β and of STG-NegEmo β vs. aggression score showed significant differences in slopes in men (*Z* = 2.70, *p* = 0.007, Figure 3B), but not in all subjects (*Z* = 1.93, *p* = 0.054, Figure 3A) or in women alone (*Z* = 0.21, *p* = 0.834, Figure 3C). However, no sex differences were found in the correlations between STG-NegEmo β and aggression score (*Z* = 1.42, *p* = 0.156).

## 4. Discussion

We showed a correlation between aggressive traits and left STG responses to monetary loss in men. Slope tests indicated that this correlation was observed in men but not women and was relatively specific to monetary loss. This finding suggests loss processing in the left STG as a specific neural correlate of aggression in men. Further, in the region-of-interest analysis, aggression did not show a significant correlation with the left STG response to identification of negative emotional faces in men, suggesting that the association may be relatively more related to monetary loss than to negative emotional face processing. However, this interpretation should be made cautiously, as the gambling and face-processing tasks differ in several psychological and methodological respects, including stimulus type, cognitive demands, and outcome contingencies. We discuss the main results below.

### 4.1. Aggression and Loss Processing

As discussed earlier, imaging studies of aggression have mostly employed behavioral paradigms that engaged participants in acts of aggression, e.g., retaliation. A meta-analysis showed that individuals with a history of aggression exhibited greater activity in the temporal cortex, including the STG, during reactive aggression (Nikolic et al., 2022). For instance, in neurotypical boys, higher aggression elicited in the ‘Point Subtraction Aggression Game’ was accompanied by activation of the ventral anterior cingulate cortex and temporoparietal junction, whereas those with attention-deficit/hyperactivity disorder and comorbid disruptive behavior disorders showed the opposite (Bubenzer-Busch et al., 2016). An event-related potential (ERP) study of a competitive reaction time task elicited aggression through provocation, where the participants set the punishment level for the opponent. A medial frontal negativity was detected in loss compared to win trials when the outcome was revealed, and this ERP was higher in magnitude in aggressive as compared to nonaggressive participants (Krämer et al., 2008). A more recent work examined proactive aggression, where participants made decisions about whether to deliver noise to interfere with the opponent’s performance. Aggression relative to nonaggression decisions engaged a wide swath of brain regions in the frontal and temporal cortices (W. Zhu et al., 2022). Most recently, in a reward–harm task, the insula, inferior frontal gyrus, inferior temporal gyrus, pallidum, and caudate showed higher activations when participants engaged in more proactive aggression under high reward expectancy (Gong et al., 2024). These studies involved social interaction, and, except for perhaps the latter work, it remained unclear how reward and/or punishment processing partakes in aggression. The current results add to this literature by suggesting relatively loss-related neural correlates of aggression in neurotypical adults.

The left STG has been consistently implicated in aggression-related neural processing. Meta-analytic evidence indicates that individuals with a history of aggression exhibit greater STG activity during reactive aggression (Nikolic et al., 2022). In antisocial men, STG activation during provocation modulates revenge-like aggressive tendencies (Weidacker et al., 2025). In healthy women, STG activation mediates the relationship between amygdala reactivity and angry faces and aggression (Buades-Rotger et al., 2016), and basolateral amygdala–STG resting-state connectivity predicts aggression (Buades-Rotger et al., 2019). These findings suggest that the left STG may serve as a hub integrating loss-related information with social-evaluative and response-preparation processes. In men with higher aggression, loss information may be assigned greater social significance, and STG responses may reflect this integrative process. Women may engage alternative processing strategies, such as emotion regulation or inhibitory control, which could explain the absence of an STG–aggression association. Future studies using loss paradigms with explicit social-evaluative components, combined with functional connectivity analyses, are needed to test this hypothesis.

### 4.2. Sex Differences in Aggression and Loss Processing

Studies have explored the neural mechanisms underlying sex differences in aggression and implicated hypothalamic-amygdala circuits in mice (Z. Zhu et al., 2024). Engaged in social reactive aggression with a modified Taylor Aggression Task, men relative to women showed higher left amygdala activation during provocation (Repple et al., 2018). Buades-Rotger and colleagues suggested that amygdala-STG resting-state connectivity anticipated an aggressive encounter and that the steroid hormone testosterone might buffer aggression by weakening this link in women (Buades-Rotger et al., 2019). Other studies highlighted the gray matter volumetrics and functional connectivities of insular subregions (Long et al., 2022) and lower fractional amplitude of low frequency fluctuation and connectivities of the left STG and supramarginal gyrus (Q. Li et al., 2020c) in the sex differences in aggression.

Here, we found that loss responses of the left STG were associated with aggression score in men but not in women, suggesting sex differences in the link of aggression and loss processing. On the other hand, it remains unclear how this finding can be meaningfully interpreted with respect to the extant literature, an issue that requires further investigation.

### 4.3. Aggression-Related STG Activity During Loss vs. Negative Emotions

Our finding suggests that the link between aggressive traits and STG-loss response does not broadly reflect negative emotion processing, as the correlation of aggression with STG response to negative emotion exposure was not significant. Nonetheless, studies have explored the relationship between negative emotions and aggression (Siep et al., 2019; Zhan et al., 2018). A study assessed testosterone, cortisol, and anger-processing neural activity effects on women’s reactive aggression, and results showed a positive relationship between amygdala reactivity to anger and aggression that was mediated by STG activation (Buades-Rotger et al., 2016). In another study, women with higher trait-level emotion regulation (ER) had a stronger resting-state connectivity between the thalamus, anterior insula and STG, but men with higher ER showed weaker connectivity (Wu et al., 2016). Thus, the relationship between negative emotion and aggression clearly needs to be investigated further.

### 4.4. Limitations

Several issues need to be considered. First, the HCP’s neurotypical sample, while sizable, limits generalizability to clinical populations (Guthman & Falkner, 2022). Second, despite extensive analyses of the task performance data, we were not able to identify a behavioral correlate of aggression. This not only limits further analyses to link the neural correlates to an objectively defined lab measure of aggression but also raises questions about the validity of the gambling task in probing the neural bases of aggression. A related issue involves the “passive” nature—guessing—of the gambling task, which may not be adequate in eliciting aggressive behavior, though it may provide a relatively “pure” measure of loss response. Further, the lack of a significant relationship between STG-loss response and aggression may simply suggest the need for a different behavioral paradigm to uncover the link in women. Third, other than task-based functional MRI, resting-state connectivity and structural metrics can inform neural correlates of aggression, both in psychopathology and as an individual trait (W. Zhu et al., 2019). Finally, irrespective of clinical, behavioral, and/or neural metrics, the relationship between loss and aggression may be confirmed with real-world data, including parsing the “tweets” of online communication (Blevins et al., 2016).

### 4.5. Conclusions

To conclude, left STG response to monetary loss was associated with higher aggression in men. This association appeared relatively loss-related, but the comparison with negative emotion processing should be interpreted cautiously because the two tasks differ in stimulus characteristics, cognitive demands, and outcome contingencies. It remains to be seen whether other behavioral paradigms may reveal the relationship between loss processing and aggression in women.

## Figures and Tables

**Table 1:** Demographics and aggression score.

Characteristic	Men (n = 473)	Women (n = 508)	t	*p*
Age (years)	27.9 ± 3.6	29.6 ± 3.6	−7.05	0.000
Education (years)	14.9 ± 1.7	15.0 ± 1.8	−1.46	0.144
Aggression score	4.1 ± 3.6	3.6 ± 3.3	2.46	0.014

## Data Availability

The data that support the findings of this study are openly available in HCP at https://db.humanconnectome.org.

## Supplementary Materials

The following supporting information can be downloaded at: https://www.mdpi.com/article/10.3390/bs16101778/s1, Figure S1: Independent anatomical ROI validation. (A) In men only, left STG response to punishment showed positive correlation with ag-gression score. (B) Scatterplot of the β estimates of left STG (STG β) vs. aggression score in men and in women; slope test confirmed the sex difference. (C) Scatter plots of STG response to punishment (STG-loss β) and to reward (STG-win β) vs. aggression score in men; (D) Left STG (yellow), flipped STG (pink), an independent anatomical left STG ROI (Anatomy3_99_lAuditory_Te11, left An99, green), and an independent anatomical right STG ROI (right An100, cyan). (E) Scatter plots of STG and of flipped STG response to punishment vs. aggression score in men. (F) Scatter plots of left An99 and of right An100 response to punishment vs. aggression score in men. slope test confirmed that this aggression-related STG response was specific to loss. Note that the residuals are plotted here with age accounted for. Table S1: Three-way repeated measures ANCOVA of RT: trial (post-win vs. post-loss) × block (reward vs. punishment) × sex, with age as a covariate. References (Wang et al., 2020; Zhang et al., 2019; Zhornitsky et al., 2019) are cited in the Supplementary Materials.
