# Supplementary material for: Sex Differences in Aggressive Traits and Superior Temporal Cortical Responses to Monetary Loss
Source: Behav Sci (Basel). 2026 Sep 29;16(10):1778. doi: 10.3390/bs16101778 (PMC13701850; doi:10.3390/bs16101778)
Supplement: Supplementary file 1 [file behavsci-16-01778-s001.zip › behavsci-4537330-supplementary.pdf]

Supplement:

Li et al., Sex differences in aggressive traits and superior temporal cortical responses to monetary loss

Supplementary Methods

Items of Achenbach Adult Self-Report (ASR)

There are a total of 120 items in 12 subscales: anxious/depressed, withdrawn, somatic complaints, thought problems, attention problems, aggressive behavior, rule-breaking behavior, intrusive behavior, other problems, as well as all critical items (items of general clinical concern), an internalizing subscale (consisting of anxious/depressed, withdrawn and somatic complaints), and an externalizing subscale (consisting of aggressive, rule-breaking, and intrusive behavior). Participants were required to circle 0 (not true), 1 (somewhat or sometimes true), or 2 (very or often true) for each item to describe themselves over the past 6 months.

Fifteen items of the aggressive behavior subscale of the Achenbach ASR

1. I argue a lot.

2. I blame others for my problems.

3. I am mean to others.

4. I get along badly with my family.

5. I get in many fights.

6. My moods swing between elation and depression.

7. I physically attack people.

8. I scream or yell a lot.

9. My behavior is very changeable.

10. I am stubborn, sullen, or irritable.

11. My moods or feelings change suddenly.

12. I have a hot temper.

13. I threaten to hurt people.

14. I get upset too easily.

15. I am too impatient.

Imaging protocol, behavioral tasks, and data preprocessing

MRI was done using a customized 3 T Siemens Connectome Skyra with a standard 32-channel Siemens receiver head coil and a body transmission coil. T1-weighted high-resolution structural images were acquired using a 3D MPRAGE sequence with 0.7 mm isotropic resolution (FOV = 224 × 224 mm, matrix = 320 × 320, 256 sagittal slices, TR = 2400 ms, TE = 2.14 ms, TI = 1000 ms, FA = 8°) and used to register functional MRI data to a standard brain space. FMRI data were collected using gradient-echo echo-planar imaging (EPI) with 2.0 mm isotropic resolution (FOV = 208 × 180 mm, matrix = 104 × 90, 72 slices, TR = 720 ms, TE = 33.1 ms, FA = 52°, multi-band factor = 8).

Participants completed two runs of a gambling task each with 4 blocks (~3 m and 12 s each run) – 2 of punishment and 2 of reward – in a fixed order (run 1: punishment – reward – punishment – reward; and run 2: reward – punishment – punishment – reward) with a fixation period (15 s) between blocks. The participants guessed whether the number of a mystery card (represented by a '?' and ranging from 1 to 9) was larger or smaller than 5 by pressing a corresponding button (Barch et al., 2013). The feedbacks comprised a green up-pointing arrow for correct guess and $1 win, a red down-pointing arrow for $0.5 loss; or a gray double-headed arrow for a wash (mystery card number = 5). The mystery number was controlled by the program and shown for 1.5 s, followed by the feedback for 1.0 s. There was a 1.0 s inter-trial interval with a "+" shown on the screen. Each block contained 8 trials. In reward blocks, 6 win trials were pseudo-randomly interleaved with either 1 neutral and 1 loss trial, 2 neutral trials, or 2 loss trials. In punishment blocks, 6 loss trials were interleaved with either 1 neutral and 1 win trial, 2 neutral trials, or 2 win trials. Thus, the amount of money won was the same across subjects.

Each participant also completed two runs of an emotion processing task each with 6 blocks – 3 of negative face and 3 of neutral shape pictures – in a fixed order: neutral – negative – neutral – negative – neutral - negative. During the emotion blocks, a target face was presented at the top of the screen and subjects were instructed to select one of two faces presented at the bottom that matched the emotion of the target face. During the neutral blocks, a target ellipse was presented at the top and subjects were to select one of two ellipses at the bottom with matching orientation. Negative faces showed angry or fearful expressions. Each block started with a cue (3 s) to indicate the current task (face or shape), followed by the stimulus (2 s) and an inter-trial interval (1 s) and, with a total of 6 trials, lasting 21 s in duration. However, as noted earlier by the HCP, the last 3 trials were missing from the emotion task because of an E-prime script bug (Barch et al., 2013).

BOLD data were analyzed with Statistical Parametric Mapping (SPM8, Welcome Department of Imaging Neuroscience, University College London, U.K.), following our published routines (Wang et al., 2020; Zhang et al., 2019; Zhornitsky et al., 2019). Images of each individual subject were first realigned (motion corrected). A mean functional image volume was constructed for each subject per run from the realigned image volumes. These mean images were co-registered with the high-resolution structural MPRAGE image and then segmented for normalization with affine registration followed by nonlinear transformation. The normalization parameters determined for the structural volume were then applied to the corresponding functional image volumes for each subject. The voxel is of 2x2x2 mm^3 after spatial normalization. Finally, the images were smoothed with a Gaussian kernel of 4 mm at Full Width at Half Maximum.

The GLM and 2nd-level analyses

Briefly, a statistical analytical block design was constructed for each individual subject, using a general linear model (GLM) by convolving the canonical hemodynamic response function (HRF) with a boxcar function in

SPM. Realignment parameters in all six dimensions were entered in the model as covariates. We constructed for each individual subject the statistical contrast “reward vs. baseline” and “punishment vs. baseline”, with baseline = 15-s fixation period between blocks in gambling task, and the statistical contrast “face vs. shape” in the emotion processing task.

Supplementary Results

In linear regression analyses (controlling for age/sex in full sample; age alone in sex-specific subgroups), RTs of punishment or reward blocks were not significantly correlated with aggressive score across all subjects (all p’s > 0.385), in men (all p’s > 0.193), or in women (all p’s > 0.469) alone. Post-loss RT or post-win RT was not correlated with aggressive score across all subjects (all p’s > 0.476), in men (all p’s > 0.120), or in women (all p’s > 0.516) alone. Thus, the extent of faster RT in post-loss or post-win trials was not related to aggressive trait. (RT of reward block) - (RT of punishment block) was not correlated with aggressive score across all subjects (r = -0.03, p = 0.402), in men (r = 0.01, p = 0.873), or in women (r = -0.06, p = 0.188) alone. (Post-loss RT of reward block) - (post-loss RT of punishment block) was not correlated with aggressive score across all subjects (r = 0.01, p = 0.791), in men (r = -0.00, p = 0.969), or in women (r = 0.02, p = 0.689) alone. Likewise, (post-win RT of reward block) - (post-win RT of punishment block) was not correlated with aggressive score across all subjects (r = -0.04, p = 0.176), in men (r = -0.02, p = 0.604), or in women (r = -0.06, p = 0.178) alone. (Post-loss RT of reward block) - (post-win RT of reward block) was not correlated with aggressive score across all subjects (r = 0.00, p = 0.990), in men (r = -0.04, p = 0.349), or in women (r = 0.05, p = 0.301) alone. Likewise, (post-loss RT of punishment block) - (post-win RT of punishment block) was not correlated with aggressive score across all subjects (r = -0.05, p = 0.104), in men (r = -0.07, p = 0.124), or in women (r = -0.04, p = 0.439) alone.

Supplementary Table S1. Three-way repeated measures ANCOVA of RT: trial (post-win vs. post-loss) × block (reward vs. punishment) × sex, with age as a covariate.

Effects	*F*	*p*
Trial	0.60	0.439
Block	9.34	0.002
Sex	25.17	0.000
Trial × Block	0.00	0.981
Trial × Sex	0.09	0.769
Block × Sex	0.12	0.726
Trial × Block × Sex	0.24	0.627

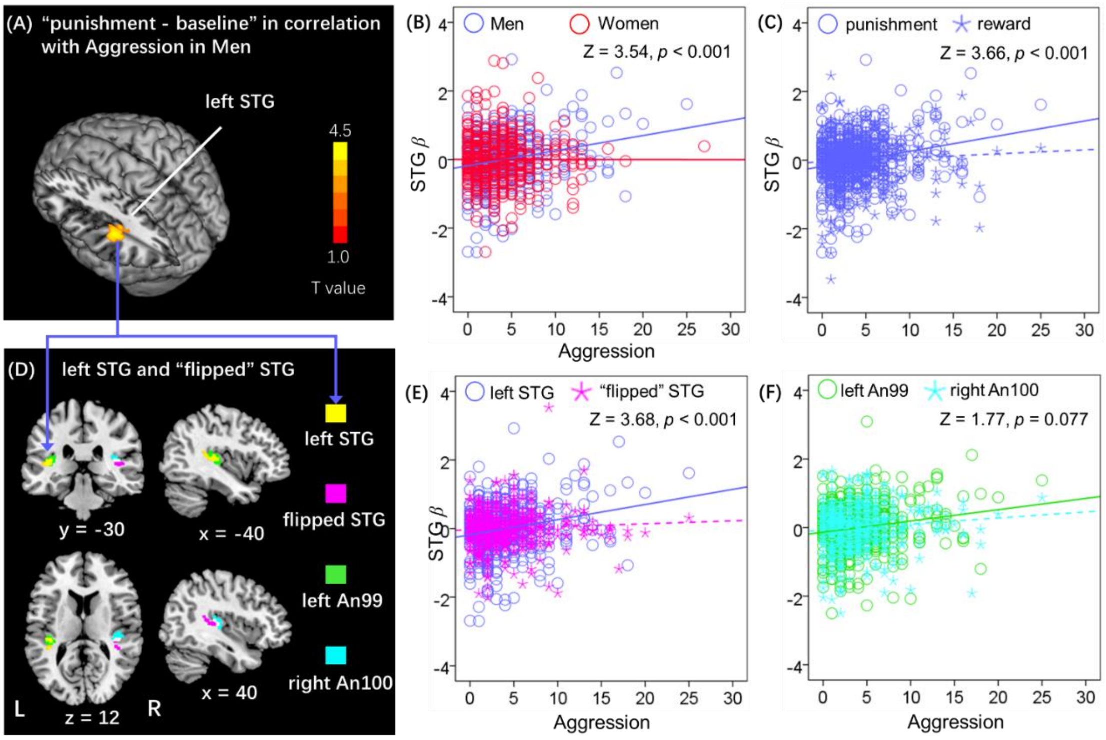

Supplementary Figure S1. Independent anatomical ROI validation. (A) In men only, left STG response to punishment showed positive correlation with aggression score. (B) Scatterplot of the β estimates of left STG (STG β) vs. aggression score in men and in women; slope test confirmed the sex difference. (C) Scatter plots of STG response to punishment (STG-loss β) and to reward (STG-win β) vs. aggression score in men; (D) Left STG (yellow), flipped STG (pink), an independent anatomical left STG ROI (Anatomy3_99_lAuditory_Te11, left An99, green), and an independent anatomical right STG ROI (right An100, cyan). (E) Scatter plots of STG and of flipped STG response to punishment vs. aggression score in men. (F) Scatter plots of left An99 and of right An100 response to punishment vs. aggression score in men. slope test confirmed that this aggression-related STG response was specific to loss. Note that the residuals are plotted here with age accounted for.

Supplementary References

Barch, D.M., Burgess, G.C., Harms, M.P., Petersen, S.E., Schlaggar, B.L., Corbetta, M., Glasser, M.F., Curtiss, S., Dixit, S., Feldt, C., Nolan, D., Bryant, E., Hartley, T., Footer, O., Bjork, J.M., Poldrack, R., Smith, S., Johansen-Berg, H., Snyder, A.Z., Van Essen, D.C., Consortium, W.U.-M.H., 2013. Function in the human connectome: task-fMRI and individual differences in behavior. NeuroImage 80, 169-189.

Wang, W., Zhornitsky, S., Le, T.M., Zhang, S., Li, C.-S.R., 2020. Heart Rate Variability, Cue-Evoked Ventromedial Prefrontal Cortical Response, and Problem Alcohol Use in Adult Drinkers. Biological Psychiatry: Cognitive Neuroscience and Neuroimaging 5, 619-628.

Zhang, S., Zhornitsky, S., Le, T.M., Li, C.R., 2019. Hypothalamic Responses to Cocaine and Food Cues in Individuals with Cocaine Dependence. Int J Neuropsychopharmacol 22, 754-764.

Zhornitsky, S., Zhang, S., Ide, J.S., Chao, H.H., Wang, W., Le, T.M., Leeman, R.F., Bi, J., Krystal, J.H., Li, C.R., 2019. Alcohol Expectancy and Cerebral Responses to Cue-Elicited Craving in Adult Nondependent Drinkers. Biol Psychiatry Cogn Neurosci Neuroimaging 4, 493-504.
